# North African Endemism: A New Species of Black Fly (Diptera: Simuliidae) from the Djurdjura Mountains of Algeria †

**DOI:** 10.3390/insects15030150

**Published:** 2024-02-23

**Authors:** Peter H. Adler, Sabrina Haouchine, Boutaïna Belqat, Abdelkader Lounaci

**Affiliations:** 1 Department of Plant and Environmental Sciences, Clemson University, Clemson, SC 29634, USA; 2 Natural Resources Laboratory, Department of Ecology and Environment, Faculty of Biological Sciences and Agronomic Sciences, Mouloud Mammeri University, Tizi Ouzou 15000, Algeria; h_saby@hotmail.fr (S.H.); lounaci@yahoo.fr (A.L.); 3 Laboratory of Ecology, Systematics, Conservation of Biodiversity (LESCB), Department of Biology, Faculty of Sciences, CNRST Labeled Research Unit N°18, Abdelmalek Essaâdi University, Tétouan 93030, Morocco; belqat@gmail.com

**Keywords:** aquatic insects, microsporidia, polytene chromosomes, *Prosimulium*, streams, taxonomy

## Abstract

**Simple Summary:**

Endemism is a hallmark of unique environments. Recognizing endemism, however, often depends on the ability to discriminate among structurally similar (cryptic) species. We used the giant polytene chromosomes of larvae to reveal a new species of black fly in the Djurdjura Mountains of northern Algeria. The female, male, pupa, and larva, in addition to the giant chromosomes, are described and compared with those of similar species. This new species is probably restricted to the mountains of the Mediterranean coast and perhaps exclusively to Algeria, making it the first endemic species of black fly recorded from the country. Recognition of this new species as endemic highlights the unique nature of the environment and provides additional rationale for conservation of aquatic habitats in the area.

**Abstract:**

Discoveries of endemic species highlight areas of biogeographic and conservation interest. Endemic species, however, are often morphologically disguised as more common and widespread species. The larval polytene chromosomes revealed a new species of black fly, *Prosimulium fungiforme*, from the Djurdjura Mountains of northern Algeria, and its female, male, pupa, and larva are described. The species is chromosomally unique; none of its 11 chromosomal rearrangements are shared with other species. Although the new species structurally resembles *Prosimulium rufipes* (Meigen) with which it previously has been confused, it can be distinguished from all other known species of *Prosimulium* in the Western Palearctic based on at least one character in each described life stage. Symbiotic organisms included two species of microsporidia, at least one of which is probably undescribed, one unknown protozoan pathogen novel in simuliids, and the trichomycete fungus *Harpella melusinae* Léger and Duboscq. Associated simuliid species included at least one new species of the genus *Helodon*. The new species of *Prosimulium* is tentatively considered endemic to the mountains of northern Algeria but might be expected in the mountains of eastern Morocco and northern Tunisia and perhaps in Sicily. If its endemic status holds, it would be the only nominal species of black fly unique to Algeria.

## 1. Introduction

Among the world’s biodiversity hotspots, mountainous regions hold some of the greatest species richness and endemism [1,2]. New endemic species continue to be discovered at a rapid pace, particularly in megadiverse taxa such as insects that inhabit biodiversity hotspots [3]. Increased geographic sampling and greater taxonomic rigor are generally responsible for these discoveries, and, in many cases, both are needed to ascertain the presence of endemic species.

The Tell Atlas Mountains, a subrange of the Atlas Mountains, run for 1500 km along the Mediterranean coast of North Africa from northeastern Morocco across Algeria into northwestern Tunisia. Except for the high Atlas Mountains, North Africa was unglaciated during the late Pleistocene [4], providing greater time for biodiversification than areas farther north that were strongly influenced by glaciation. Numerous endemic insects inhabit this maritime mountain chain [5,6,7,8]. The largest massif in the Tell Atlas is the somewhat centrally located Djurdjura Mountain range in Algeria, with peaks rising to 2300 m above sea level. Several endemic insects have been reported from the Djurdjura range [9,10], and more might be expected, given, for instance, that Djurdjura National Park, situated within the Djurdjura range, has 36 endemic vascular plant taxa [11]. Algeria is second in water scarcity of all countries in Africa, making it especially vulnerable to climate change and anthropogenic insults [12]. As environmental threats increase, surveys of Algeria’s flora and fauna, particularly in freshwater habitats, become increasingly urgent.

North Africa, with its wide variety of landscapes and climates [13], has a rich fauna of black flies (Simuliidae). It is the southernmost area for five simuliid genera [14]: *Greniera* Doby & David, *Helodon* Enderlein, *Metacnephia* Crosskey, *Prosimulium* Roubaud, and *Urosimulium* Contini. Best represented among these genera in North Africa is *Prosimulium*, with four species, all in the *P. hirtipes* species group [14]. Given the extensive Tell Atlas chain and other mountain ranges, we would expect not only more representatives of these genera but also endemic species. A total of 6 endemic black flies, all in the genera *Helodon* and *Simulium* Latreille, have been found in Morocco, but none of the 33 nominal species in Algeria—1.4% of the world’s 2398 described species—are endemic [14]. The black flies in the Tell Atlas range of Algeria, however, have not been taxonomically studied, with the exception of one chromosomal investigation of the *Simulium aureum* group [15]. The Djurdjura Mountains have been sampled for black flies, but morphological identifications revealed only widespread species and taxonomic studies have not been conducted [16,17,18].

The banding patterns of the giant polytene chromosomes have been instrumental in revealing hidden biodiversity and evolutionary relationships in the family Simuliidae, and because they are typically unique to a species, they have been used analogously to a barcode in the description of numerous new species of black flies in conjunction with morphological and molecular analyses [19]. The giant chromosomes of more than 570 species of black flies have been described [20], facilitating comparative studies. These chromosomal barcodes are particularly important for uncovering cryptic species of black flies in morphologically uniform taxa, such as the *Prosimulium hirtipes* group [21], which has received little molecular attention but for which there is a substantial database of species-level chromosomal information [22,23].

We describe a new species of black fly in the *P. hirtipes* species group from the Djurdjura range of Algeria based on the chromosomal band patterns and morphological characters of the female, male, pupa, and larva. We also examine the relationships of this new species—the 34th nominal species in the family Simuliidae recorded from Algeria—and discuss its possible endemism.

## 2. Materials and Methods

Larvae in the Djurdjura Mountains of Algeria were collected into 3 changes of 1:3 acetic ethanol (Carnoy’s fixative [24]) in 2015 and 2016; associated pupae and several larvae were collected into 80% ethanol (Table 1).

### 2.1. Chromosome Preparation

Slide preparations of polytene chromosomes from the larval silk glands, plus one gonad for sex determination, were prepared according to the Feulgen staining procedure [22]. Chromosomal band sequences were compared against the standard sequence for the genus *Prosimulium* [22,23,25].

Representatives of all six chromosome arms were photographed with a Jenoptik ProgRes^®^ SpeedXT Core 5 digital camera on a BH-2 Olympus microscope. Chromosomal maps were constructed from digital images using Adobe^®^ PhotoShop^®^ (Version 8.0) Elements 8.

New inversions in the long (L) and short (S) arms of each chromosome (I, II, and III) were numbered following the last number used in each arm in the genus *Prosimulium* [21]. Fixed inversions are italicized in the text and on the maps; polymorphic inversions are not. Deleted bands (del) are indicated by the chromosome arm and section number (e.g., IIL del59). Section numbers on the chromosome maps reflect the standard sequence for the genus *Prosimulium* [22,23,25].

### 2.2. Morphological Preparation

Specimens of each life stage above the egg were dissected in 80% ethanol. The antenna and venter of the larval head capsule, pupal gill, and pharate adult antennae, palps, and tergite X with cerci were removed and temporarily slide-mounted in a drop of 50% acetic acid under a coverslip. The dorsum of the larval head capsule was cleared in cold 10% potassium hydroxide and transferred to glycerin in a depression slide. Pharate adult terminalia were heated for ca. 2 min in 85% lactic acid, moved to a drop of cold 85% lactic acid in a depression slide, further dissected into component parts, and oriented for interpretation and imaging [21].

Photographs of structures were taken at multiple focal planes with a Jenoptik ProgRes^®^ SpeedXT Core 5 digital camera mounted on an Olympus BX40 light microscope. Helicon Focus (version 7.7.5) stacking software was used to form composite images from multiple focal planes. All morphological images were made from specimens collected at the type locality.

Morphological procedures and terminology follow standard practice typically used for the Simuliidae [24] and presented briefly here. Pharate adults in ethanol, after dissection from the pupal case, were chemically dried using hexamethyldisilazane and pinned with a minuten through the thorax. The pupal case and cocoon were placed in a microvial with glycerin and pinned through the stopper beneath the associated adult. Dissected parts were pinned in a separate microvial beneath each adult. Descriptions of hair colors were based on pinned specimens; colors of body parts were not described except in general terms because of the incompletely sclerotized pharate condition of the specimens, which renders colors paler. Similarly, hind leg measurements were not taken because of the naturally bent condition in the pupal casing.

Larvae with patent infections of pathogens were prepared by placing a drop of infected tissue in a drop of 50% acetic acid and applying a coverslip; one microsporidum-infected larva was Feulgen-stained. The guts of 3 penultimate-instar larvae at site 3 (10 May 2015) were dissected out, the gut contents removed, the peritrophic matrix and hind gut placed in a drop of 50% acetic acid, a coverslip applied, and the preparation examined for trichomycete fungi. All larvae in Carnoy’s fixative were transferred to 80% ethanol after chromosomal preparation [24].

### 2.3. Type Depository

The holotype and paratypes are deposited in the U.S. National Museum of Natural History (USNM) in Washington, DC. Additional paratypes are deposited in the Clemson University Arthropod Collection.

## 3. Results

***Prosimulium fungiforme*** Adler, Belqat, and Haouchine, new species

*Prosimulium rufipes*: [16,17] not Meigen (misident.)

*Prosimulium rufipes*: [18] not Meigen (misident.); *ruffoi*: incorrect spelling, page 150

### 3.1. Chromosomal Description

**Figure 1:**
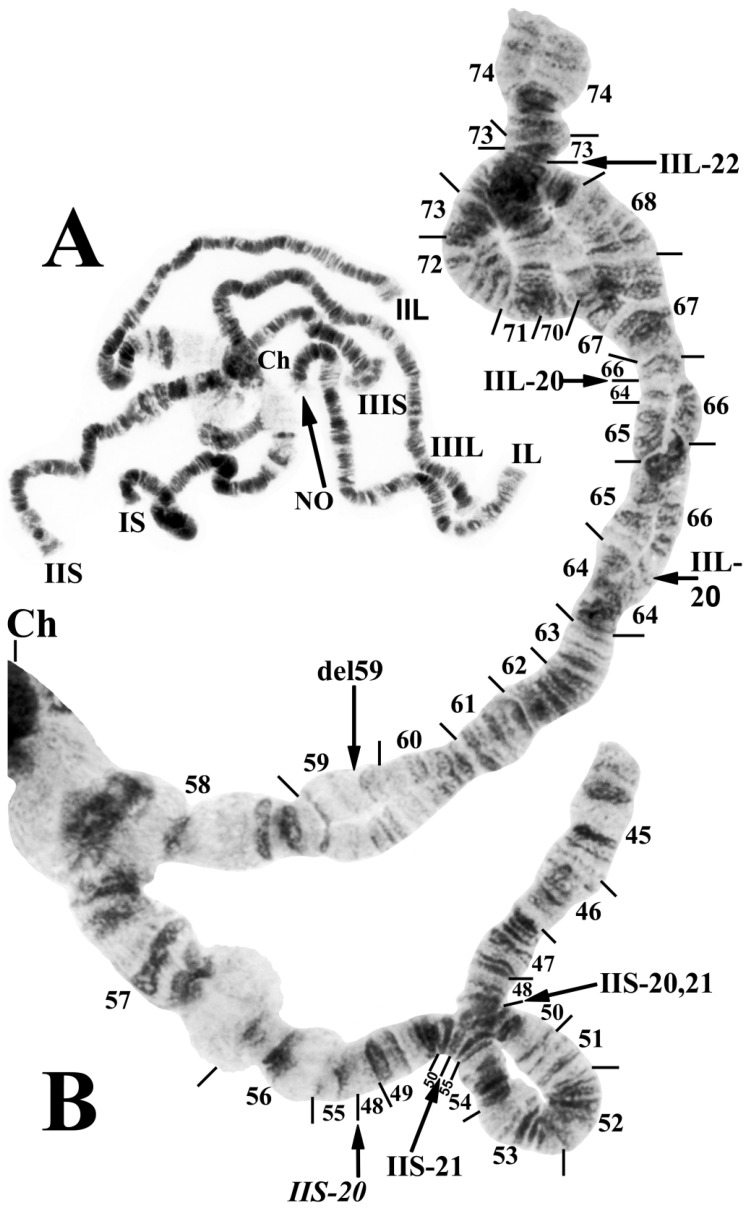
Chromosomes of *Prosimulium fungiforme* from Algeria. (**A**) Entire complement (female larva) with arms radiating from chromocenter (Ch); chromosome arms (IS, IL, IIS, IIL, IIIS, and IIIL) are indicated; NO, nucleolar organizer. inverted segments indicated by arrows. (**B**) Chromosome II, the sex chromosome, of *Prosimulium fungiforme* (male larva), showing the complex XY sequence heterozygous for IIS-21 (on top of fixed inversion *IIS-20*), IIL-20, 21, 22, and deleted band del59 (arrow). The distal break of Y-linked IIL-22 is indicated with an arrow, and the complex looped section between section 67 and the middle of section 73 results from heterozygosity of IIL-21 and IIL-22. Sections 57–58 represent the expanded centromere region of chromosome II.

**Figure 2:**
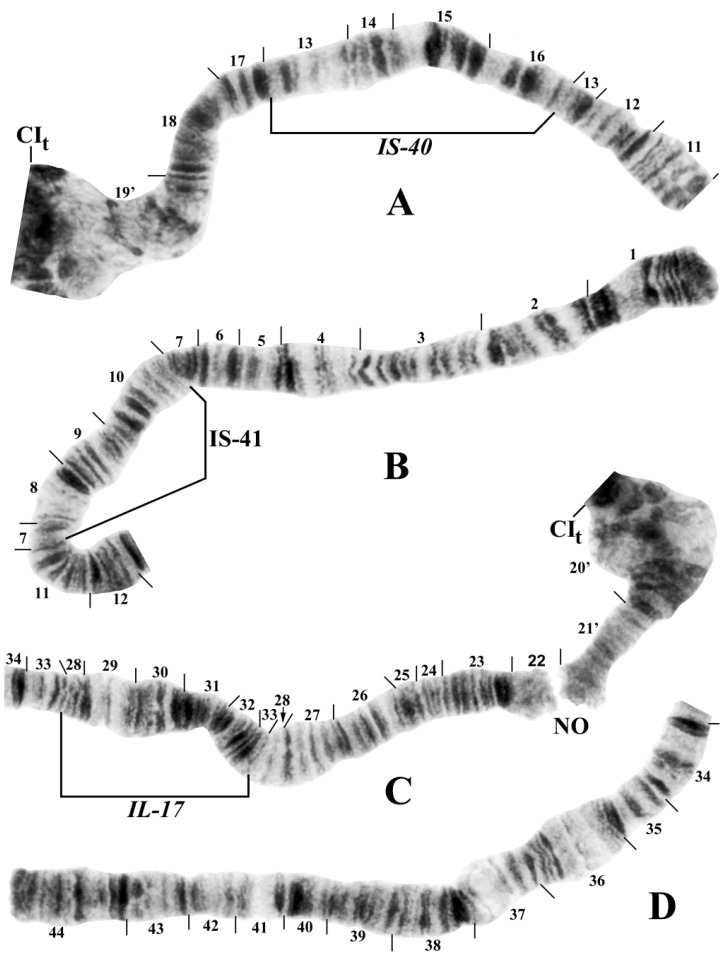
Chromosome I (female larva) of *Prosimulium fungiforme* from Algeria (inverted segments bracketed). (**A**) Basal half of IS, including the transformed centromere (CI_t_) region, showing the *IS-40* sequence. (**B**) Distal half of IS showing the sequence of the autosomal IS-41 polymorphism. (**C**) Basal half of IL showing the *IL-17* sequence; NO, nucleolar organizer. inverted segments indicated by arrows. (**D**) Distal half of IL, standard sequence.

**Figure 3:**
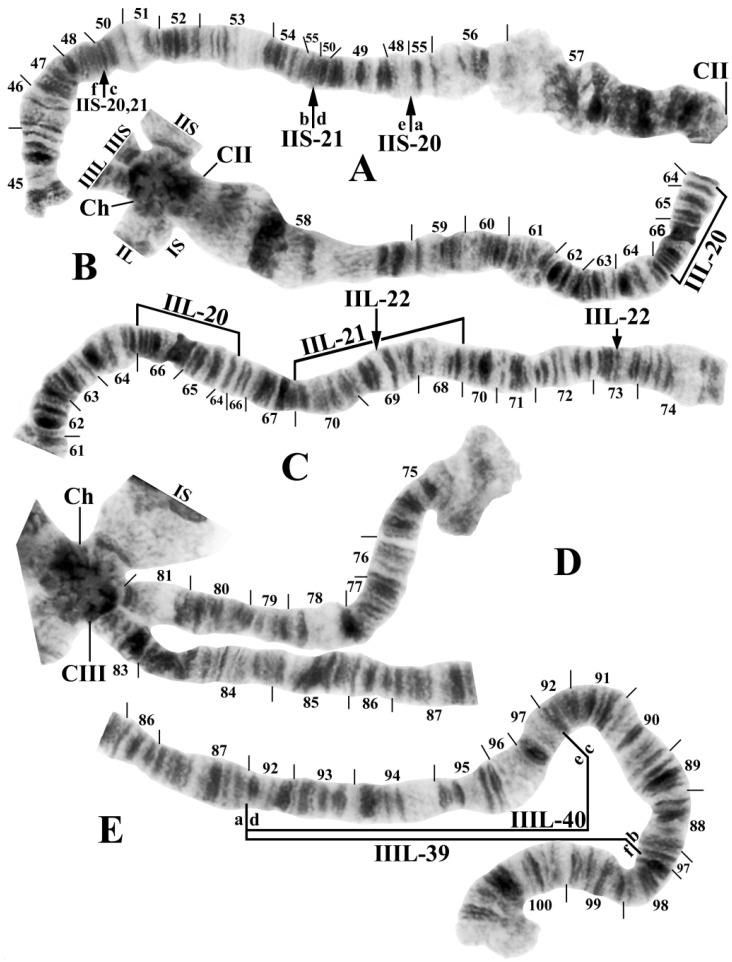
Chromosomes (female larvae) of *Prosimulium fungiforme* from Algeria (inverted segments bracketed or indicated by arrows). (**A**) IIS showing the *IIS-20* sequence with the IIS-21 X-linked inversion superimposed; arranging the letters a–f will yield the standard *Prosimulium* sequence; CII, centromere of chromosome II. (**B**) Basal half of IIL showing the centromere (CII), attachment of all arms (IS, IL, IIS, IIL, IIIS, and IIIL) to the chromocenter (Ch), and IIL-20 X-linked sequence. (**C**) Distal half of IIL showing the IIL-20, 21 X sequence; breakpoints of Y-linked IIL-22 (not present) are indicated by arrows. (**D**) IIIS standard sequence and base of IIIL, both emanating from the chromocenter (Ch) and attached by the centromere (CIII). (**E**) IIIL showing the *IIIL-39*, *40* sequence; arranging the letters a–f will yield the standard *Prosimulium* sequence.

The polytene banding sequences of 41 larvae (30 females and 11 males) revealed a new species of *Prosimulium*, distinguished by a chromocenter, 5 fixed rearrangements, 1 autosomal polymorphism, and complex sex chromosomes (Table 2; Figure 1, Figure 2 and Figure 3).

All larvae had the typical haploid number of three chromosomes and standard arm associations. The chromosomal homologues were tightly (>90%) paired, the nucleolar organizer was in the standard location (chromosome section 22) for the genus *Prosimulium* (Figure 1A and Figure 2C), and the terminus of IIIS was flared (Figure 1A and Figure 3C). The centromere region of chromosome I was transformed (sensu [23]), and that of chromosome II (sections 57–58) was expanded (Figure 1B and Figure 2A,C). The centromeres of all three chromosomes were attached to a darkly staining chromocenter (Figure 1A and Figure 3B,D).

Only chromosome arm IIIS had the standard banding sequence for the genus *Prosimulium*; all other arms carried at least one unique fixed inversion (IS, IL, IIS, and IIIL) or sex-linked rearrangement (IIL). IS, IL, and IIS were fixed for one inversion each: *IS-40* (Figure 2A), *IL-17* (Figure 2C,D), and *IIS-20* (Figure 3A), respectively. IIIL had extra heterochromatin in the base, followed by two fixed overlapping inversions: *IIIL-39* and *IIIL-40* (Figure 3D,E).

IIL was the sex chromosome, bearing a complex set of three X-linked inversions involving both the short and long arms (IIS-21, IIL-20, and IIL-21) and one Y-linked inversion (IIL-22) (Figure 1B and Figure 3A–C). A thin band in section 59 of IIL was deleted in one homologue of a male larva (Figure 1B) from site 4, although whether it was linked to the X or Y could not be determined because of tight homologue pairing between it and the nearest sex-linked inversion.

Only one autosomal inversion, IS-41 (Figure 2B), was observed, and it was in high frequency (>0.85/site).

### 3.2. Morphological Description

**Figure 4:**
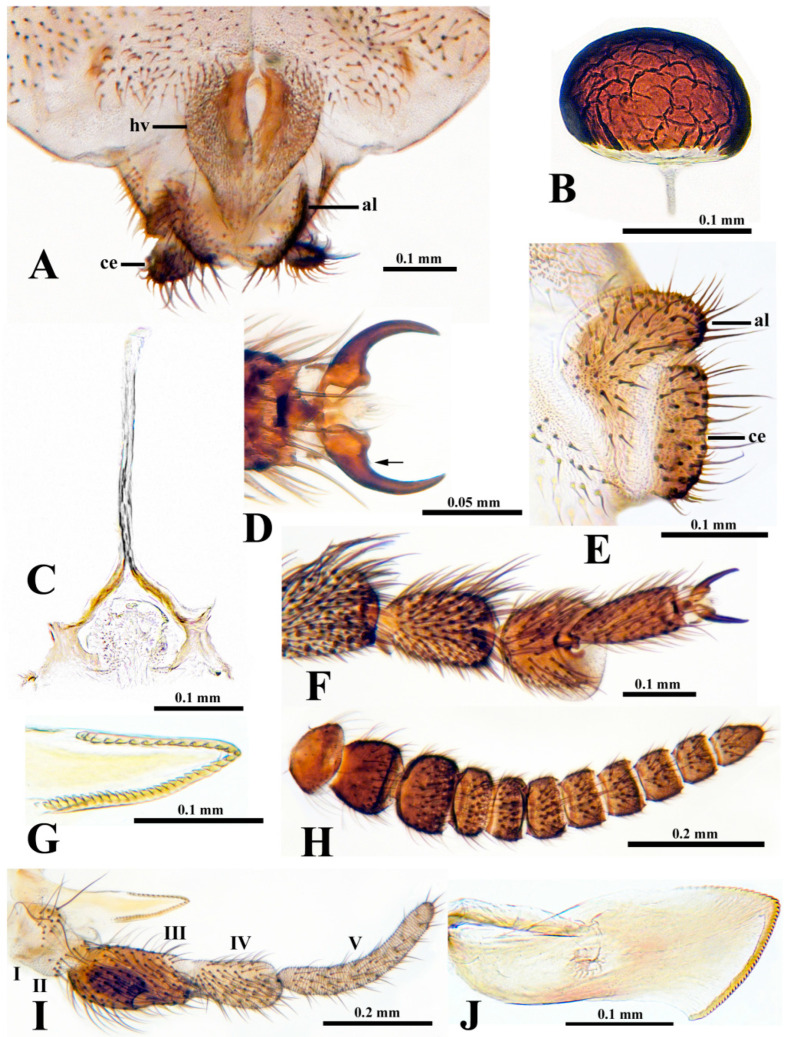
Female of *Prosimulium fungiforme* from Algeria. (**A**) Terminalia and portion of sternite VIII, highlighting hypogynial valves (hv), anal lobes (al), and cerci (ce) (ventral view). (**B**) Spermatheca. (**C**) Genital fork. (**D)** Claws of hind leg; arrow indicates minute sub-basal peg. (**E**) Anal lobe (al) and cercus (ce) (lateral view), both labeled at posterior margin. (**F**) Tarsomeres I (apex only) through IV and claws of hind leg. (**G**) Lacinia (apex). (**H**) Antenna. (**I**) Maxillary palp and lacinia; palpomeres indicated by Roman numerals. (**J**) Mandible.

**Female** (*n* = 2, except as otherwise indicated). Thorax length 1.6 mm. Body dark brown. All hair pale golden. Head about 0.7 times as wide as thorax. Frons and clypeus entirely haired; frons 0.4 times as wide as head. Labrum and clypeus subequal in length. Antenna (Figure 4H) with scape, pedicel, and nine flagellomeres; proportional lengths of pedicel, first flagellomere, and second flagellomere 1.4:1.4:1.0 (*n* = 1); pedicel about 0.9 times as wide as first flagellomere 0.9. Maxillary palp (*n* = 1) (Figure 4I) with proportional lengths of third, fourth, and fifth palpomeres 2.0:1.0:1.4; sensory vesicle (Figure 4I) ovoid, about 0.5 times length of third palpomere, with small, round mouth. Lacinia (Figure 4G,I) with 39 retrorse teeth (*n* = 1). Mandible (Figure 4J) with 36 inner teeth and 16 outer teeth (*n* = 1). Pleural membrane, katepisternum, and postnotum bare. Precoxal bridge incomplete. Legs brown; coxa through tibia paler than remainder of legs. Hind leg without calcipala and pedisulcus. Claw (Figure 4D,F) a curved talon, unarmed except for a minute sub-basal peg (arrow in Figure 4D). Wing with costa, subcosta, and radius bearing fine setae dorsally and ventrally. Abdominal segment VIII with small sclerotized sternal plate; other segments lacking sclerotized sternal plate. Hypogynial valves (ovipositor lobes) (Figure 4A) gently curved toward midline, weakly sclerotized except inner margin of each valve; inner margins concave, creating teardrop-shaped space. Genital fork (Figure 4C) with stem and arms slender, weakly sclerotized; space between arms mitre-shaped; each arm expanded into subquadrate lateral plate, produced at each corner. Anal lobe in lateral view (Figure 4E) narrowed along anterior margin, expanded posteroventrally as broadly rounded lobe extended to posterior margin of cercus. Cercus in lateral view (Figure 4E) short, subrectangular, about 2.7 times as wide as long. Spermatheca (Figure 4B) mushroom-like, about 1.6 times wider than long, wrinkled in reticulate pattern, heavily pigmented except broad basal area completely devoid of pigment; spermathecal duct and both accessory ducts unpigmented.

**Figure 5:**
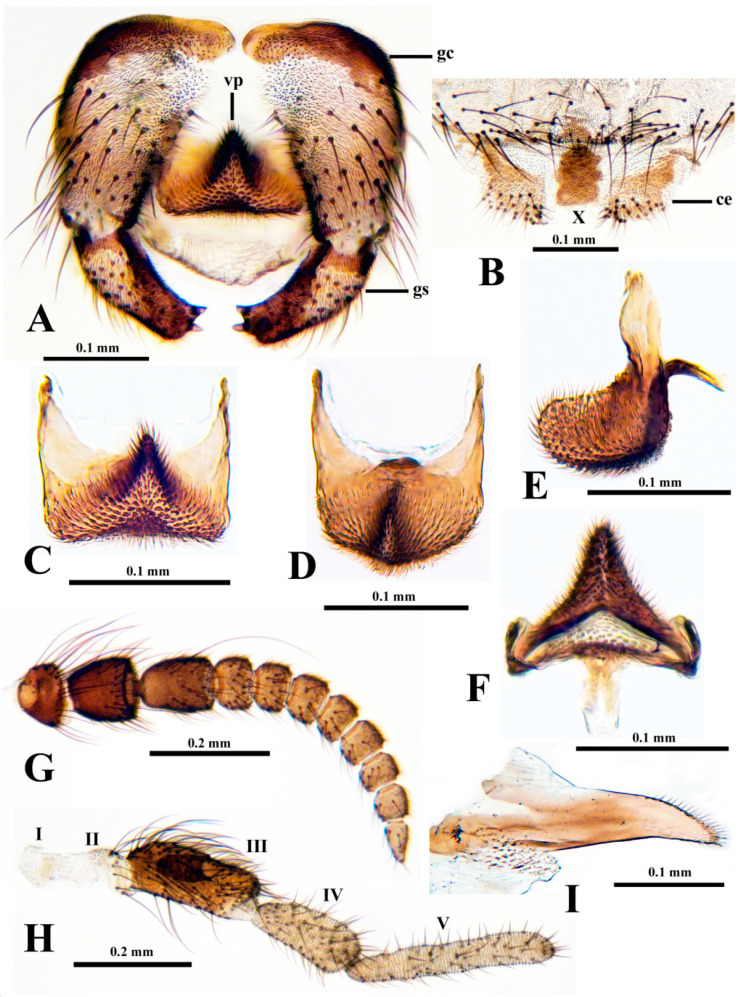
Male of *Prosimulium fungiforme* from Algeria. (**A**) Genitalia (ventral view); gc, gonocoxa; gs, gonostylus; vp, ventral plate. (**B**) Posterior of tergite IX, tergite X, and cerci (ce) (dorsal view). (**C**–**F**) Ventral plate. (**C**) Ventral view. (**D**) Ventral view, tilted. (**E**) Lateral view. (**F**) Terminal view. (**G**) Antenna. (**H**) Maxillary palp; palpomeres indicated by Roman numerals. (**I**) Lacinia.

**Male** (*n* = 1). Thorax length 1.5 mm. Body generally dark brown to matte black. Hair pale golden on dorsum of thorax, coppery elsewhere. Head 0.65 times as wide as thorax. Antenna (Figure 5G) brownish, with scape, pedicel, and nine flagellomeres; proportional lengths of pedicel to first flagellomere 1.0:1.0 and widths 1.1:1.0. Maxillary palp (Figure 5H) with proportional lengths of third, fourth, and fifth palpomeres 1.2:1.0:1.8; sensory vesicle (Figure 5H) about 0.4 times as long as third palpomere, with small, round mouth. Lacinia (Figure 5I) with about 30 apical and subapical hairs. Katepisternum, pleural membrane, and postnotum bare. Legs brownish, except portions of femora and tibiae paler. Hind leg without calcipala and pedisulcus. Wing with costa and radius bearing fine setae. Gonocoxite in ventral view (Figure 5A) about 1.2 times longer than gonostylus. Gonostylus in ventral view (Figure 5A) smoothly curved toward midline, gradually tapered, with two apical spinules. Ventral plate in ventral view (Figure 5A,C,D) subrectangular, with posterolateral corners rounded and posterior margin slightly concave to convex, depending on angle of tilt; minute setae in broad triangular pattern; arms parallel to one another, forming broad U-shape; in lateral view (Figure 5E) well rounded, with blunt apex; in terminal view (Figure 5F) subtriangular, slightly concave laterally. Median sclerite (Figure 5F) short, bifurcated apically. Paramere subquadrate, with slender anterior projection and rounded edges, without spines or setae. Dorsal plate absent. Aedeagal membrane with fine setae. Abdominal tergite X (Figure 5B) minute, about 1.4 times longer than wide (slide-mounted). Cercus (Figure 5B) minute, with 21–24 setae.

**Figure 6:**
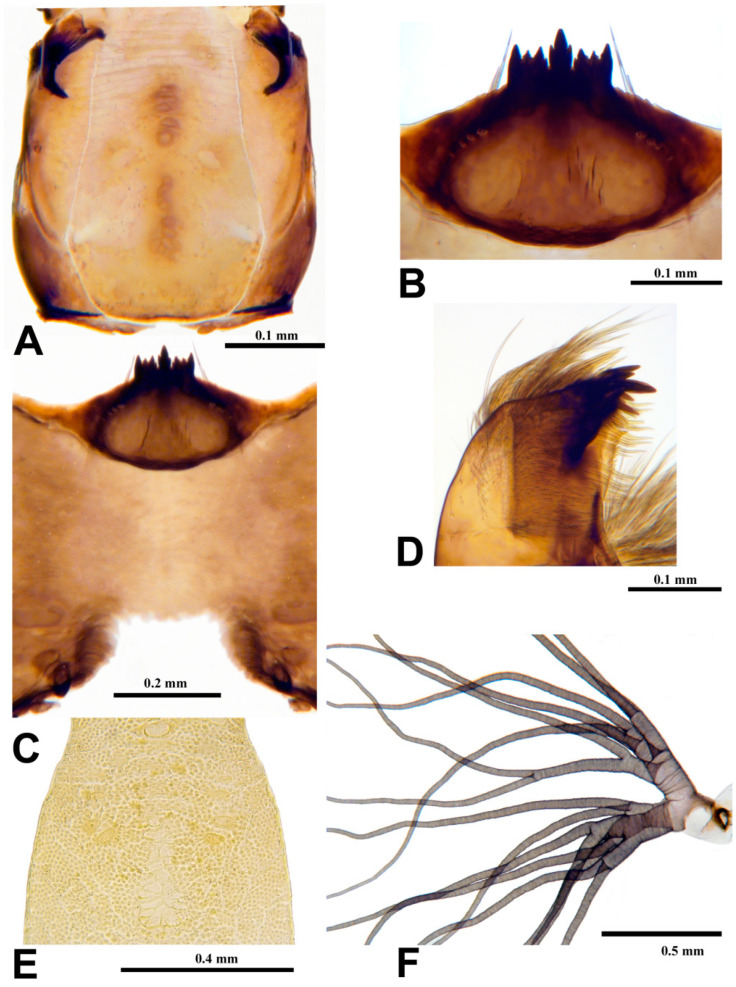
Larva and pupa of *Prosimulium fungiforme* from Algeria. (**A**) Head capsule (dorsal view). (**B**) Hypostoma (ventral view). (**C**) Head capsule (ventral view, subesophageal ganglion removed during clearing). (**D**) Mandible (apex, aboral surface). (**E**) Cephalic plate of male pupa (truncated anteriorly and posteriorly). (**F**) Pupal gill (lateral view, apices truncated).

**Pupa** (*n* = 14). Length (excluding gill) 3.9–4.8 mm, mean = 4.3 mm. Cephalic plate (Figure 6E) smooth but with dense pattern of minute, rounded, barely raised microtubercles, and one pair of unbranched facial trichomes near antennal base; clypeal plate with two pairs of unbranched trichomes per side. Thorax with faint, superficial transverse wrinkles; cuticle with dense covering of minute, rounded, barely raised microtubercles; three or four unbranched dorsal trichomes per side, plus pair of unbranched trichomes posterior to gill base laterally and trio of unbranched trichomes per side immediately lateral to cephalic plate near antennal base. Gill (Figure 6F) about 0.3–0.5 times as long as pupa, with 16 slender, grayish filaments in 3 groups arising from short basal stalk about as long as wide; stalks of all three groups about as long as four times longer than width; branching pattern: dorsal group with eight filaments arranged as (2 + 1 + 2) + (1 + 2) [parentheses indicate shared petioles], lateral and ventral groups each with two petiolate pairs of filaments; petioles of three groups splayed in nearly same plane such that gill opens basket-like in frontal view; filaments furrowed. Abdomen densely covered with minute, slightly raised, rounded microtubercles, dorsally with postscutellar bridge bearing four small unbranched setae per side; segment I with four–six small unbranched setae per side; segment II with six–eight small unbranched setae per side; segments III and IV each with four recurved hooks and two small unbranched setae per side; segments V–IX each with anterior spine comb and two–four small unbranched setae per side; segment IX with pair of long, slender terminal spines and eight moderately long, unbranched setae. Abdomen laterally with pleurites on segments IV and V large, subquadrate, each bearing two small unbranched setae per side; pleurites on segments VI and VII small, slender, elongate, without setae; striate membrane with zero–three small unbranched setae per side. Abdomen ventrally with segment III unarmed; segment IV bearing pair of slender hooks and two or three small unbranched setae per side; segments V–VII each with pair of stout, bifid or trifid hooks and at most one small unbranched seta per side. Cocoon sac-like, without definitive structure, densely woven, typically covering pupa.

**Mature larva** (*n* = 10). Length 7.1–8.5 mm, mean = 8.1 mm. Body (in Carnoy’s fixative) pale grayish. Head capsule (Figure 6A) orangish brown to chestnut brown; anteromedial and posteromedial head spots brown, forming a central line; anterolateral and posterolateral spots negative, often vague, sometimes with pale brown pigment along their borders. Venter of head capsule (Figure 6C) with horizontal long spot and round spot on each side of postgenal cleft brown; subesophageal ganglion pigmented, pale gray. Antenna about as long as labral fan stalk, with basal and medial articles hyaline and distal article dark brown; proportional lengths of proximal, medial, and distal articles (not including cone sensillum) 1.0:1.4:1.0. Labral fan with 33–39 (mean = 36.0) primary rays. Mandible (Figure 6D) with 3 large, dark distal teeth (3rd from apex largest, most apical tooth slightly larger than 2nd) followed proximally by 3 moderately dark teeth (3rd largest followed by 1st), numerous spinous teeth (sensu [26]), and 11–15 (mean = 13.6) marginal teeth. Hypostoma (Figure 6B) with median tooth extended anteriorly beyond all other teeth; sublateral teeth posterior to lateral teeth and extended to same level as tines of median tooth, with five or six lateral serrations and four sublateral setae per side. Postgenal cleft (Figure 6C) short, with anterior margin straight or slightly biarctate, 0.2–0.3 times as long as postgenal bridge (measured from anterior margin of anterior tentorial pits to hypostomal groove). Cervical sclerites (Figure 6A) minute, enclosed within occiput. Gill histoblast of 16 long, thread-like filaments. Lateral plate of thoracic proleg well-sclerotized, slender, L-shaped. Abdominal cuticle with sparsely scattered, colorless, unbranched setae, about six–eight times length of more common, minute, colorless setae. Rectal papillae of three finger-like lobes. Anal sclerite subrectangular, with anterior and posterior arms subequal in length; anterior arms broadened distally into less sclerotized cuticle with clusters of minute, slender, brown spines; posterior arms darkly sclerotized, narrowed distally. Posterior circlet with 81–84 rows of 10–14 hooklets per row.

### 3.3. Diagnosis

Chromosomally, *P. fungiforme* can be distinguished from all known black flies by any of its five fixed inversions (*IS-40*, *IL-17*, *IIS-20*, *IIIL-39*, and *IIIL-40*) or its complex sex chromosomes involving both arms of chromosome II. The female is distinguished from other members of the *P. hirtipes* species group in the Western Palearctic by the mushroom-shaped, rather than elongated, spermatheca. The male has a slightly wider ventral plate than other members of the *P. hirtipes* group in the Mediterranean Basin. The pupa differs from other species of the *P. hirtipes* group in the Mediterranean Basin by the 16 gill filaments that open into a basket-like arrangement, rather than 14 to 27 filaments in a tight or splayed cluster. The larva can be distinguished from other species of Western Palearctic *Prosimulium* by the head-spot pattern that consists of a central line of brown anteromedial and posteromedial spots and vague, negative anterolateral and posterolateral head spots, rather than all spots typically positive and brownish.

### 3.4. Type Material

Holotype female (dissected from pupal case and pinned with exuviae and cleared abdomen in glycerin vials, USNM). ALGERIA, below Tirurda Pass, 1 km upstream from a drinking water fountain called “L’ɛinser n’biya” (TR3), 36°29′36′′ N 04°21′18′′ E, elevation 1255 m, collected by S. Haouchine, 16 April 2015.

Paratypes: ALGERIA, same data as holotype (5 pupae, 2 pupal exuviae, 48 larvae including 14 originally collected into ethanol); 24 April 2015 (1 female and 1 male [both dissected from pupal cases and pinned with exuviae], 11 pupae). Stream 1 km downstream of Tirurda Pass (TR1), 36°29′26′′ N 04°21′42′′ E, elevation 1120 m, collected by S. Haouchine, 7 April 2015, (1 pupa); 16 April 2015 (1 pupa with pharate male, 2 larvae); 10 May 2015 (29 larvae). Spring brook 1.2 km upstream of village Tirurda (TR2), 36°29′26′′ N 04°21′32′′ E, elevation 1045 m, collected by S. Haouchine, 7 April 2015 (35 larvae). Stream near town of Illithen, 36°30′25′′ N 4°24′17′′ E, elevation 1150 m, collected by S. Haouchine, 14 April 2016 (18 larvae); 27 May 2016 (16 larvae). Stream near village of Ath Atsou, 36°29′43′′ N 04°22′23′′ E, elevation 1080 m, collected by S. Haouchine, 2 April 2016 (3 larvae); 14 April 2016 (44 larvae).

### 3.5. Bionomics

*Prosimulium fungiforme* was collected in the Djurdjura Range about 45 km south of the Mediterranean coast. The immature stages inhabited torrential headwater streams at elevations above 1000 m, with rocky substrates, maximum widths of about 1.5 m, and maximum temperatures of about 18 °C (as *P. rufipes* [18]). The type locality is a stream originating from springs and small streams fed by rainwater and melting snow from Tirurda Pass (ca. 1700 m above sea level). The stream at the type locality dries out for a variable period between June and November. A new species of leptophlebiid mayfly, *Habrophlebia djurdjurensis* Kechemir, Sartori, and Lounaci, was collected and described from some of the same sites where *P. funigforme* was taken [10].

### 3.6. Associated Species

Simuliids associated with *P. fungiforme* included two species in the *Simulium vernum* group, a species of the *S. argenteostriatum* group, and, at Assif Illithen (14 April 2016), an undescribed species of the genus *Helodon*, here designated *Helodon* ‘species X’. This undescribed species resembles *H. laamii* (Beaucournu-Saguez and Bailly-Choumara), known only from the Rif Mountains of Morocco, but differs in the number of terminal gill filaments on each of the 3 large, tubular gill bases: 3, 4, and 5 (total 12) in ‘species X’ versus 2, 2, 1 (total 5) in *H. laamii*. Chromosomal examination of two female larvae of ‘species X’ confirms its placement in the genus *Helodon*. The complement shows the IIS–IIIL IIIS–IIL whole-arm interchange characteristic of all chromosomally studied species [20,27] of *Helodon*. Unlike all other chromosomally studied species of *Helodon*, which have the nucleolar organizer in the center of IIL [27], this undescribed species has the nucleolar organizer in the centromere region of chromosome I. Additional rearrangements in most of the arms are also present, relative to the standard band sequence [28] for *Helodon*. The location of the nucleolar organizer in chromosome I is perhaps not unexpected, given the extraordinary geographic gap between the typical northern Holarctic distribution of *Helodon* and the two North African species of the genus. The nearest location of a northern *Helodon* species is nearly 3000 km to the north of ‘species X’.

### 3.7. Natural Enemies

**Figure 7:**
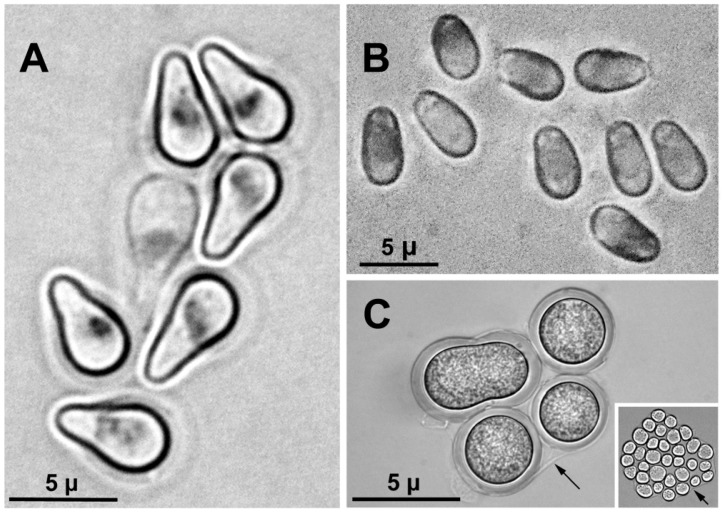
Pathogens from larvae of *Prosimulium fungiforme* from Algeria. (**A**) Feulgen-stained spores of an unknown species of microsporidium from male larva, showing stained nucleus in each spore. (**B**) Unknown species of microsporidium (unstained) from larva, taken with stage diaphragm partially closed. (**C**) Unknown protozoan pathogen from larva. The inset shows a cluster of entities, far less frequent than the typical larger entities in the image, within a thin envelope. Scale bar is the same for the inset; arrows in both images indicate a thin envelope.

Three larvae (one at site 1 and two at site 2) had heavy microsporidian infections that manifested in the fat body of the abdomen as dense white asymmetric masses of spores and other microsporidian developmental stages. The pyriform spores, each about 5 µ long (Figure 7A), matched the shape and length of those of a microsporidian species, probably undescribed, known only from larvae of *Simulium petersoni* Stone and DeFoliart in Utah [24]. The microsporidian from *P. fungiforme*, however, is unlikely to be conspecific with the microsporidian in *S. petersoni*, given the probability of cryptic species of microsporidia [24] and the disparate taxonomic affinities (*Prosimulium* vs. *Simulium*) and biogeographic regions (Palearctic vs. Nearctic) of the host. A second microsporidian species (Figure 7B) infected a larva at site 5. The outward pathology resembled that of larvae infected by the microsporidium with pyriform spores, but the 5 µ long spores were oval.

An additional pathogen, probably a protozoan (*cf*. *Entamoeba*), infected the abdominal hemocoel of one larva at site 4 (Figure 7C). The infection affected larval development; the larva was larger than the histoblasts of the thoracic gill and appendages indicated, and the abdomen was largely devoid of fat body characteristic of all other larvae at the site. The abundant spherical bodies in the abdomen moved freely in the hemocoel when the cuticle was pushed with forceps. Under a light microscope, these spherical bodies were surrounded by a conspicuous, thick, translucent envelope (perhaps a cyst wall). Occasionally, two or more of these entities were within a thinner envelope; clusters of more than 30 smaller bodies could be seen within a thin envelope (Figure 7C, inset). A pathogen with these characteristics has not heretofore been reported among the many symbiotes known from black flies.

One additional symbiote was discovered in larvae of *P. fungiforme*. The peritrophic membrane of larvae at site 3 was colonized by the trichomycete fungus *Harpella melusinae* Léger and Duboscq. This obligatory fungus of simuliids is nearly cosmopolitan, although it is probably a complex of species [29].

### 3.8. Etymology

The species name *fungiforme* is from the Latin for “mushroom” and “form” in reference to the mushroom-shaped spermatheca of the female, an unusual shape for the organ in the *P. hirtipes* species group, in which it is typically oval (i.e., longer than wide).

## 4. Discussion

Despite morphological similarity with numerous other species of black flies, an analysis of the giant polytene chromosomes of specimens from Algeria’s Djurdjura Mountains unequivocally revealed a chromosomally distinct species. *Prosimulium fungiforme* is chromosomally unique, sharing no inversions with any other known simuliid species. *IIS-20*, however, is deceptively similar to *IIS-3* of several members of the Palearctic *P. hirtipes* group [22,23,30], differing only by the inclusion of a small group of fine bands in section 48. Of the other rearrangements in *P. fungiforme*, a chromocenter is present in myriad simuliids, but it typically evolves independently in species [27]. In the genus *Prosimulium*, it has no phylogenetic value, although it does have species-level diagnostic importance for *P. fungiforme*. No other chromosomally known species of the *P. hirtipes* group in the Palearctic Region [22,23,30] has a large, true chromocenter. The European species that have been described as having a weak or strong “chromocentric tendency” in some nuclei [23] are more appropriately considered to express ectopic pairing of centromeres, rather than having a mass of additional heterochromatin typical of a true chromocenter to which all centromeres in a nucleus attach. The transformed centromere region in chromosome I and expanded centromere region in chromosome II are shared with all chromosomally known members of the *P. hirtipes* group in the Old World [22,23].

The new species has complex sex chromosomes involving both arms of chromosome II. Sex-linked chromosomal rearrangements in the Simuliidae are typically restricted to one arm. Less frequently, sex linkage involves both arms, typically as pericentric inversions; of 65 species evaluated, 18.7% of the sex-linked inversions were pericentric as opposed to paracentric [31]. The least frequent case, known in two species of *Stegopterna* in western North America [32] and in *P. fungiforme*, is for both arms to have sex-linked paracentric inversions exclusive of the centromere.

Although the chromosomal complement of *P. fungiforme* is highly rearranged, the associated morphological features deviate little from those of most other members of the *P. hirtipes* species group, particularly *P. rufipes* (Meigen), with which it has been confused in previous reports (i.e., [16,17,18]). The new species is also structurally similar to *P. calabrum* Rivosecchi of southern Italy but is chromosomally quite remote from it; the chromosomes of *P. calabrum* are similar to those of *P. rufipes* studied by P.H.A. Structural changes are not necessarily correlated with macrogenomic (i.e., chromosomal) changes. The pupal gills of some species in the *P. hirtipes* group, for instance, are unique but are associated with minimal chromosomal differentiation [22]. Presumably, these structural changes are associated with microgenomic (i.e., molecular) rearrangements.

The most conspicuous diagnostic structural feature of *P. fungiforme* is the mushroom-shaped spermatheca, which contrasts with the typical oval form in nearly all other members of the *P. hirtipes* species group [24]. The mushroom form, in which the spermatheca is wider than long, is diagnostic for the *P. magnum* species group [24]. The spermatheca can have diagnostic value at the generic and species levels, but it has infrequently been used in descriptions or illustrations of Palearctic black flies. The illustrated exceptions for the *Prosimulium hirtipes* group include a few European species [33,34] and the Far Eastern *P. jezonicum* ((Matsumura) [35]; as *P. hirtipes*), each of which has the typical oval spermatheca, longer than wide. *Prosimulium isos* Rubtsov of Siberia, however, appears to have a spermatheca wider than long [33], warranting a further study of the spermatheca across the entire *P. hirtipes* group. Other morphological characters diagnostic for the *P. hirtipes* group (e.g., male gonostylus with two, rather than three–seven, apical spinules and larval abdomen rather abruptly expanded at segment V, rather than gradually expanded) [24] are present in *P. fungiforme*, and alone or in conjunction with the chromosomal banding pattern unambiguously confirm the placement of this species in the *P. hirtipes* group.

A caveat in comparative morphology of species in the Palearctic *P. hirtipes* group is that available treatments are limited and often based on outdated taxonomic understanding (e.g., [33,36]). Further complicating any comparisons are the disparate orientations of terminalia in the literature, particularly for males. A slight tilt of a genitalic component in any direction can change the shape (and generate a putative new species). Accordingly, orientation of structures is of paramount importance for description, illustration, and imaging. The limitations outlined for comparative morphology of the *P. hirtipes* group underscore the need for an integrated taxonomic approach. Without the benefit of chromosomal analysis, *P. fungiforme* might have continued to go unnoticed as a species distinct from *P. rufipes*. Limited taxon sampling of the *P. hirtipes* group in molecular analyses further underscores the need for chromosomal analysis.

*Prosimulium fungiforme* has not been found in the extensively sampled Rif Mountains of Morocco [37] nor in chromosomally sampled areas of Mediterranean Europe, such as Spain and Italy. No species of *Prosimulium* has been discovered in Sardinia [14]. We note, however, that *P. rufipes*, which *P. fungiforme* superficially resembles, has been recorded from Sicily on morphological grounds [38]. *Prosimulium rufipes*, however, is a species of more northern, non-Mediterranean areas of the Palearctic Region [22]. A chromosomal investigation of putative *P. rufipes* in Sicily is warranted. The Moroccan species formerly known as *P. rufipes* in the literature is probably an undescribed species, chromosomally more similar to *P. rachiliense* Djafarov [14]. *Prosimulium fungiforme* might be present in other areas of the Tell Atlas range, perhaps in eastern Morocco and northern Tunisia. The illustrations of putative *P. rufipes* from Tunisia [39], however, suggest that they are not of the new species; the larval head capsule shows dark, rather than pale (negative) anterolateral head spots. We do not expect that *P. fungiforme* will be found beyond the Tell Atlas range. We also suspect that one or more of the pathogens we discovered in the larvae will have distributions largely congruent with *P. fungiforme* and, therefore, would also be endemic. The discovery of another species of black fly (*Helodon* ‘species X’) known only from the Djurdjura range suggests that the potential for simuliid endemicity, and that of simuliid symbiotes, has not been fully explored. Given the propensity of cryptic species in the Simuliidae, we expect that at least some of the anticipated endemism of simuliids in the Djurdjura range and the larger Tell Atlas will probably involve cryptic species, a situation also seen in baetid mayflies [8]. The new species of simuliids provide further evidence of the unique biodiversity of the Djurdura Mountains and its aquatic habitats, supporting the need for conservation and protection of the area’s freshwater environment.

## Figures and Tables

**Table 1:** Collection sites for *Prosimulium fungiforme* in Algeria.

Site	Location	Longitude and Latitude	Elevation (m above Sea Level)	Date
1	spring brook 0.5 km upstream of village Tirurda (TR2)	36°29′26′′ N 04°21′32′′ E	1045	7 April 2015
2	stream 1 km upstream from a drinking water fountain called “l3insar [*sic*] n’biya” (TR3)	36°29′36′′ N 04°21′18′′ E	1255	16 April 2015
3	stream 1 km downstream of Tirurda Pass (TR1)	36°29′26′′ N 04°21′42′′ E	1120	10 May 2015
4	stream near town of Illithen	36°30′25′′ N 04°24′17′′ E	1150	14 April 2016
5	stream near village of Ath Atsou	36°29’43′′N 04°22′23′′ E	1080	14 April 2016

**Table 2:** Frequency of chromosomal inversions in *Prosimulium fungiforme* from Algeria, relative to the *Prosimulium* generic banding sequence.

Site ^1^	1	2	3	4	5
Females:Males	6:0	10:4	2:1	9:3	3:3
*IS-40*	1.00	1.00	1.00	1.00	1.00
IS-41	0.92	0.86	1.00	0.88	1.00
*IL-17*	1.00	1.00	1.00	1.00	1.00
*IIS-20*	1.00	1.00	1.00	1.00	1.00
IIS-21 ^2^	*	*	*	*	*
IIL-20 ^2^	*	*	*	*	*
IIL-21 ^2^	*	*	*	*	*
IIL-22 ^3^	**	**	**	**	**
IIL del59 ^4^				***	
*IIIL-39*	1.00	1.00	1.00	1.00	1.00
*IIIL-40*	1.00	1.00	1.00	1.00	1.00

^1^ Site numbers correspond to collection data in Table 1. ^2^ An asterisk (*) indicates IIS-21, IIL-20, and IIL-21 were linked to the X chromosome. ^3^ A double asterisk (**) indicates IIL-22 was linked to the Y chromosome. ^4^ A triple asterisk (***) indicates a deleted (del) band in one male larva, although whether it was on the X or Y chromosome could not be determined.

## Data Availability

All data supporting reported results are included in the text.
